# The Transcriptomic Landscape of Age-Induced Changes in Human Visceral Fat and the Predicted Omentum-Liver Connectome in Males

**DOI:** 10.3390/biomedicines11051446

**Published:** 2023-05-14

**Authors:** Diogo de Moraes, Felippe Mousovich-Neto, Sarah Santiloni Cury, Jakeline Oliveira, Jeferson dos Santos Souza, Paula Paccielli Freire, Maeli Dal-Pai-Silva, Marcelo Alves da Silva Mori, Geysson Javier Fernandez, Robson Francisco Carvalho

**Affiliations:** 1 Department of Structural and Functional Biology, Institute of Biosciences of Botucatu, Sao Paulo State University, UNESP, Botucatu 18618-689, SP, Brazil; 2 Department of Biochemistry and Tissue Biology, University of Campinas, Monteiro Lobato St., 255, Campinas 13083-862, SP, Brazil; 3 Obesity and Comorbidities Research Center (OCRC), University of Campinas, Campinas 13083-862, SP, Brazil; 4 Experimental Medicine Research Cluster (EMRC), University of Campinas, Campinas 13083-862, SP, Brazil; 5 Grupo Biologia y Control de Enfermedades Infeciosas (BCEI), Facultad de Ciencias Exactas y Naturales, Universidad de Antioquia (UdeA), Medellín 050010, Colombia

**Keywords:** visceral adipose tissue, aging, obesity, fat-liver crosstalk, age-related diseases

## Abstract

Aging causes alterations in body composition. Specifically, visceral fat mass increases with age and is associated with age-related diseases. The pathogenic potential of visceral fat accumulation has been associated with its anatomical location and metabolic activity. Visceral fat may control systemic metabolism by secreting molecules that act in distal tissues, mainly the liver, through the portal vein. Currently, little is known about age-related changes in visceral fat in humans. Aiming to identify molecular and cellular changes occurring with aging in the visceral fat of humans, we analyzed publicly available transcriptomic data of 355 omentum samples from the Genotype-Tissue Expression portal (GTEx) of 20–79-year-old males and females. We identified the functional enrichment of genes associated with aging, inferred age-related changes in visceral fat cellularity by deconvolution analysis, profiled the senescence-associated secretory phenotype of visceral adipose tissue, and predicted the connectivity of the age-induced visceral fat secretome with the liver. We demonstrate that age induces alterations in visceral fat cellularity, synchronous to changes in metabolic pathways and a shift toward a pro-inflammatory secretory phenotype. Furthermore, our approach identified candidates such as *ADIPOQ-ADIPOR1/ADIPOR2, FCN2-LPR1*, and *TF-TFR2* to mediate visceral fat-liver crosstalk in the context of aging. These findings cast light on how alterations in visceral fat with aging contribute to liver dysfunction and age-related disease etiology.

## 1. Introduction

Aging induces significant changes in body composition [1,2]. In general, a decline in fat-free mass in parallel to an increase in fat mass and ectopic fat deposition occurs in mammals as a consequence of aging [1,3]. Body fat is redistributed across adipose tissue depots during aging, with the accumulation of fat in the abdominal region being positively correlated with age, as reflected by anthropometric indicators such as waist-to-hip ratio and waist circumference [4]. Redistribution of body fat to the abdominal region with age is a matter of clinical relevance, given the association between abdominal fat accumulation, mortality, cardiometabolic diseases [5], and frailty [6]. These features are all associated with aging [7], indicating that abdominal fat mass and function may represent a key determinant of health and lifespan outcomes during aging.

In the abdominal region, fat is stored in two different compartments: subcutaneous adipose tissue (aSAT) and visceral (intra-abdominal) adipose tissue (aVAT). Studies in humans strongly suggest that fat accumulation in both abdominal compartments correlates positively with metabolic disturbances in men and women. In particular, the aVAT has been shown to have a stronger correlation with metabolic disorders than aSAT. Moreover, the aVAT content is more effective than the body mass index and the waist circumference ratio for predicting cardiovascular risk [8]. Several factors hint toward the association between visceral adiposity and metabolic disease risk. Remarkably, the portal vein drains intraperitoneal adipose tissues, such as mesenteric and omental (both aVAT), thereby having a direct connection to the liver. In addition to their different anatomical location, adipocytes in the aVAT are considered to be different from those in the aSAT in terms of their developmental origin, adipokine secretion profile, size and metabolic activity [9].

The role of aVAT in disease pathogenesis has been extensively explored in experimental models. For instance, surgical removal of visceral adipose tissue (VAT) increases rodent median and maximum lifespans and promotes insulin sensitivity [10]. Moreover, artificially inducing visceral obesity by transplanting VAT into the abdominal cavity causes liver insulin resistance in mice, and such an effect is abolished when visceral fat from interleukin-6 knockout mice is engrafted into the recipient mice [11]. These and other findings support the portal vein theory, which postulates that visceral fat secretes molecules that reach the liver through the portal vein, resulting in hepatic steatosis, insulin resistance, and metabolic dysfunction [12]. Hence, given the relevance of the visceral fat as well as the visceral fat-liver crosstalk in determining disease risk, investigating the impact of aging on VAT and its communication with the liver may provide valuable insights into the pathophysiology of age-associated metabolic diseases.

Despite the extensive knowledge available regarding the role of adipose tissue in the pathophysiology of obesity and metabolic disturbances, much less is known about the underlying consequences of aging on adipose tissue function. This gap has begun to be filled by studies carried out on rodents [13,14,15,16,17]. However, in humans, only a few studies have addressed the impact of aging on adipose tissue. Of note, these studies have gathered initial information with regard to how aging changes the subcutaneous adipose tissue (SAT) [18,19,20,21]. Strikingly, as far as we know, no studies have explored how aging impacts visceral fat in humans with high throughput sequencing. Indeed, it is a challenge to obtain samples for a powered study design aiming to elucidate age-induced alteration in visceral fat, mainly due to the high invasiveness required for visceral fat sampling in humans.

Here, we aimed to functionally explore the gene expression landscape of the human visceral adipose tissue during aging and expand the portal vein theory by predicting novel ligands and their respective receptors, which we call omentum-liver age-associated connectome. By doing so, we proposed hallmarks of VAT aging and established potential visceral fat-liver crosstalk contributing to the onset of age-related diseases.

## 2. Methods

### 2.1. Subjects and Dataset

Transcriptome data of omentum visceral adipose tissue (GTEx v7) of 241 males (*n* = 14 for 20–29 years old, *n* = 18 for 30–39 years old, *n* = 32 for 40–49 years old, *n* = 93 for 50–59 years old, *n =* 75 for 60–69 years old, *n =* 9 for 70–79 years old) and 114 females (*n* = 11 for 20–29 years old, *n =* 10 for 30–39 years old, *n =* 25 for 40–49 years old, *n =* 31 for 50–59 years old, *n =* 34 for 60–69 years old, *n =* 3 for 70–79 years old) were downloaded along with the available clinical data. Trimmed mean of m-values (TMM) (*n =* 13 for 20–29 years old, *n =* 17 for 30–39 years old, *n =* 28 for 40–49 years old, *n =* 83 for 50–59 years old, *n =* 58 for 60–69 years old, *n =* 7 for 70–79 years old) and 114 females (*n =* 10 for 20–29 years old, *n =* 8 for 30–39 years old, *n =* 23 for 40–49 years old, *n =* 30 for 50–59 years old, *n =* 33 for 60–69 years old, *n =* 3 for 70–79 years old) normalized expression tables were downloaded directly from the GTEx website (https://www.gtexportal.org/, accessed on 20 January 2020). There were fewer samples in the TMM normalized expression table as samples present in BioJupies were added at different times. 

The sample size for male liver samples was 161 (*n =* 4 for 20–29 years old, *n =* 14 for 30–39 years old, *n =* 24 for 40–49 years old, *n =* 60 for 50–59 years old, *n =* 56 for 60–69 years old, *n =* 3 for 70–79 years old) and 65 for females (*n =* 3 for 20–29 years old, *n =* 3 for 30–39 years old, *n =* 11 for 40–49 years old, *n =* 23 for 50–59 years old, *n =* 23 for 60–69 years old, *n =* 2 for 70–79 years old).

### 2.2. Differential and Time-Dependent Gene Expression Analyses

Differential expression analyses were performed on BioJupies [22] using limma [23] with raw counts normalized to log10Counts per million (CPM), that is, dividing each sample by the total sum of its counts, then multiplying them by one million and applying log10-transform, using cutoffs of FDR < 0.05 and FC ≥ 2.0 for at least one of the comparisons. We used participants of the age rang 20–29 years old as common references. This procedure was repeated twice using either only males or females. Time-dependent expression analysis in visceral fat was performed in two steps: differential expression and clusterization. Our analysis was adapted from other overtime expression analyses [24,25]. The main difference is that, instead of comparing each group with the rest, we have used the earliest time point as a common reference. K-means clustering (K = 11) was performed on Morpheus (https://software.broadinstitute.org/morpheus (accessed on 20 January 2020)) with mean normalized expression. To compare the average expression of males and females and visualize the tendency of each cluster, TMM expression was normalized in (0, 1) for each sex and cluster. Arithmetic averages of all normalized genes were plotted. The area under the curve (AUC) was calculated using Prism (v8.00 for Windows, GraphPad Software, La Jolla, CA, USA, www.graphpad.com, accessed on 20 January 2020) and the AUC ratio between men and women was established. This method was adapted from previous reports [24,25].

### 2.3. Secretome Analysis

All differentially expressed genes (DEGs) were classified as secreted or not secreted based on the Human Protein Atlas database (http://www.proteinatlas.org (accessed on 20 January 2020); v18proteinatlas.org/humanproteome/tissue/secretome accessed on 20 January 2020) [26,27]. 

### 2.4. Enrichment Analyses

Functional enrichment analyses were performed on Enrichr [28], a web-based tool that integrates various enrichment libraries. Results from KEGG, GO Cellular component, GO Biological Process, and GO Molecular Function were extracted, and only significant enrichments were included (FDR < 0.05) [29]. Terms that were unlikely relevant in this context (e.g., toxoplasmosis infection, Chagas disease) were manually removed from the figures but can be found in Supplementary Materials. Because co-expressed genes are likely functionally similar [30], enrichment analysis was performed on whole clusters. Enrichment scores were log-transformed and multiplied by −1. 

### 2.5. Cell Signature Analysis

We generated cell signature scores with XCell [31].Gene expression data (transcripts per million; TPM) were downloaded from the GTEx website, and because XCell requires heterogeneous datasets for better performance, data from aortic tissue, whole blood, transformed lymphocytes, and transformed fibroblasts from GTEx were included along with omental adipose tissue data. To test whether the scores increased or decreased with aging, one-way ANOVA test for trend analysis was performed using Prism (v8.00 for Windows, GraphPad Software, La Jolla, CA, USA, www.graphpad.com, accessed on 20 January 2020).

### 2.6. Liver Connectome Analysis

Ligands were selected based on the following criteria: an expression of ≥25 TPM in at least 25% of all visceral fat samples (GTEx v8, 541 samples); considered as secreted by the Human Protein [26]; presence in clusters A, B, C or I and at least one predicted [32] with one of the receptors found in liver. Liver receptors were selected based on the same criteria (GTEx V8, 226 samples) except for secretion and cluster status. Both ligands and receptors were queried at Enrichr [28], and KEGG enrichments [29]. Only enrichments with FDR < 0.05 were retrieved. The age and sex of liver samples used in the analysis were distributed as follows: for males, 4 samples from 20–29 years old, 14 from 30–39 years old, 24 from 40–49 years old, 60 from 50–59 years old, 56 from 60–69 years old, and 3 from 70–79 years old; and for females, 3 samples from 20–29 years old, 3 from 30–39 years old, 11 from 40–49 years old, 23 from 50–59 years old, 23 from 60–69 years old, and 2 from 70–79 years old. We performed a manual curation to rule out false positives based on the criteria that the secreted protein must be found in the blood but not in other compartments such as the extracellular space based on The Human Protein Atlas [33].

### 2.7. Genome-Wide Association Study (GWAS) Overlap Analysis

Ligands and receptors from the omentum-liver connectome found in the previous analyses were queried in GWAS Catalog [34] and the GeneAge [35]. Associated phenotypes were selected based on their relative relevance to the current study, such as body mass index, adiposity, insulin sensitivity, aging, longevity, etc.

### 2.8. Gene Network Analysis

Gene network analysis was performed in the STRING [36] database and the connections were based on text mining, coexpression, and experiments. The figures were edited using Cytoscape [37]. The eigenvector centrality of the networks was calculated using the CytoNCA plugin for Cytoscape [38].

## 3. Results

### 3.1. Transcriptome Alterations Induced by Aging in Visceral Fat Unravel Altered Cellularity and Increased Inflammation

In total, we observed 708 DEGs in males and 102 DEGs in females, present in at least one age range group. In males, we identified DEGs starting from 40–49 years. On the other hand, in the samples obtained from women, we observed DEGs at later ages—i.e., 50 years old or older (Figure S1). Next, having set DEGs for each age group in both women and men, we clustered these DEGs through k-means clustering. By doing so, we obtained 11 clusters of genes (Figure S2). Four of these clusters revealed genes whose expression changed gradually (Figure S3, clusters A, B, C, and I). Compared with other clusters or randomly generated gene sets, their Protein-protein interaction enrichment *p*-values were lower (Table S1), indicating they are likely to ex-ercise biological function.

**Figure 1:**
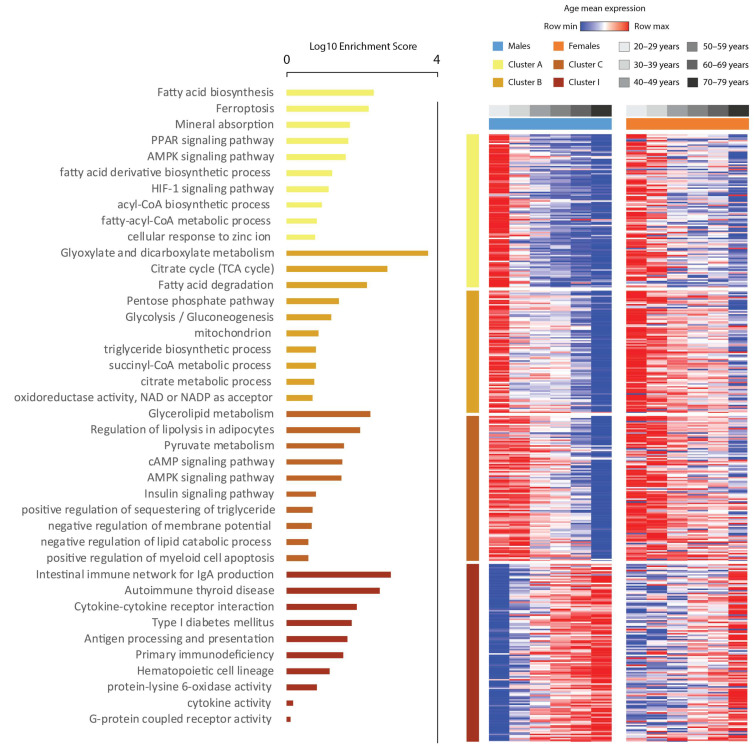
Heatmap of the mean expression of differentially expressed genes (DEGs) in at least one age group of both men and women. These genes were clustered for k-means = 11, and only clusters that formed gradients were displayed. The figure shows which genes become more expressed with age (cluster I) and less expressed with age (clusters A, B, and C). The functional enrichment of clusters A, B, and C indicates an overall decrease in genes involved in lipid metabolism, adipocyte differentiation, and energy expenditure processes, while the expression of genes involved in inflammatory and type I diabetes mellitus pathways increases. These genes are differentially expressed in at least one age group compared to the youngest group used as a reference. Row values are normalized by Z–score, so minimum and maximum values are row relative.

**Figure 2:**
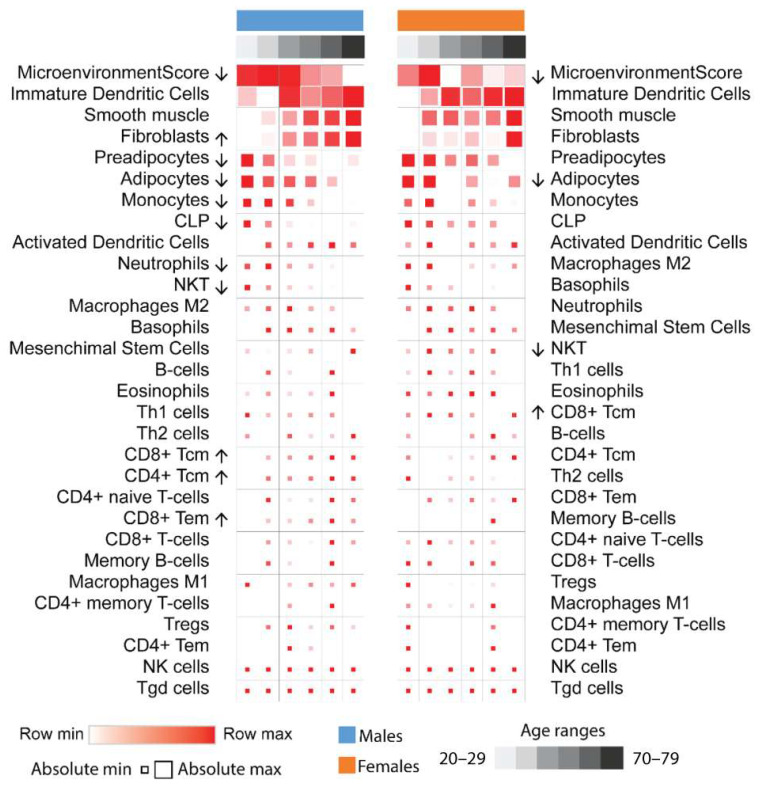
Cellular deconvolution analysis. Mean cell signature scores of each age range were calculated using XCell, which estimates cell-type populations based on transcriptomics data. Arrows indicate significance (*p* < 0.05) for trends with aging according to the one–way ANOVA test. Adipocyte, preadipocyte, monocyte, natural killer cell (NKT), and neutrophil populations decrease with age in males, while fibroblast and some lymphocyte populations increase. These patterns were similar in females but were not significant. Row values are normalized by Z-score, so minimum and maximum values are row relative and reflected by color. Absolute nonrelative values are reflected by square size.

In both sexes, gene expression in clusters A, B, and C decreased, while expression in cluster I increased with aging. “Lipid metabolism”, “adipocyte differentiation”, and “lipid droplets” terms were enriched in clusters A, B, and C, respectively, while “Cytokine-cytokine receptor interaction”, “Antigen processing and presentation”, “Cytokine activity”, and “Type I diabetes mellitus” terms were enriched in cluster I (Figure 1). These data suggest a decrease in the adipogenic potential and adipocyte number in parallel with changes in the immunological landscape of the adipose tissue driven by aging. To obtain further insight regarding the enrichment analysis, we performed cell type deconvolution analysis [31]. Here, cell type proportions also varied in different age ranges (Figure 2). Immature dendritic cells had the highest overall scores, followed by fibroblast, preadipocytes, and adipocyte scores. Mean fibroblast scores increased significantly with age in males, but with only a tendency to increase in females, while adipocyte scores decreased significantly in both. Preadipocyte scores also decreased significantly in males, with a tendency to decrease in females. Monocytes and natural killer cell scores decreased significantly in males, while some classes of CD4 and CD8 T cells increased, albeit their scores were very low among all samples.

We also aimed to elect genes that could be the central mediators of these changes by gene–gene interaction network analysis. Eigenvector centrality revealed fatty acid synthase (*FASN*), stearoyl-CoA desaturase (*SCD*), and diacylglycerol-O-acyltransferase 2 (*DGAT2*) as the most central genes for clusters A, B, and C (Figure S4a). CD40 ligand (*CD40LG*), interferon gamma (*IFNG*), interferon gamma-induced protein 10 (*CXCL10*) and C-X-C motif chemokine receptor 4 (*CXCR4*) were the most central in cluster I. This means that these genes were connected to other highly connected genes in these hubs by being coexpressed or through physical protein–protein interactions (Figure S4b). To simplify the networks, we selected the most central genes in each cluster and verified their interaction. This revealed that the interaction of *IFNG* and adiponectin (*ADIPOQ*) bridged the networks (Figure S4c).

**Figure 3:**
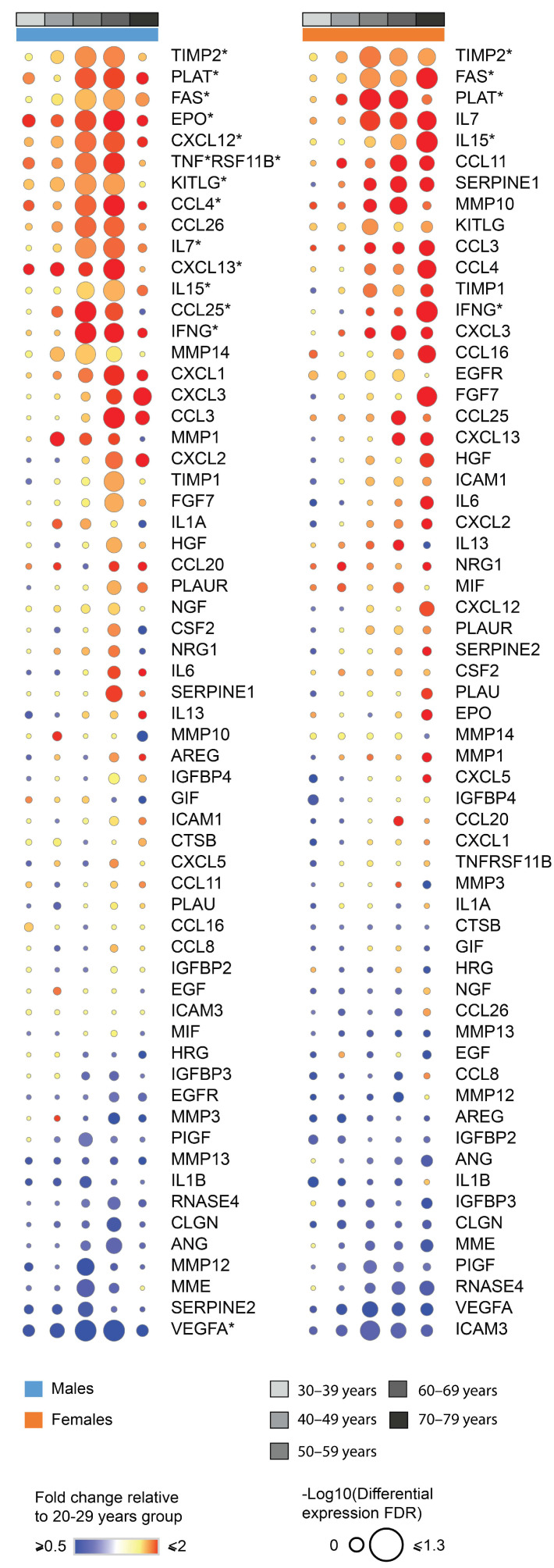
Fold change and false discovery rate of genes corresponding to the senescence-associated secretory phenotype (SASP). Genes marked with * have FDR < 0.05 for limma differential expression in at least one age group. This was obtained with differential expression analysis, using the youngest group as a reference. This figure is a general overview of the profile of SASP, including those genes that were not found to be differentially expressed.

As senescent cells have a specific senescence-associated phenotype (SASP) and accumulate along with aging within the adipose tissue [39], we sought to verify whether SASP-related genes would be affected by aging within adipose tissue. Indeed, we found changes in the expression of multiple SASP-related genes in males (*TIMP2*, *PLAT*, *FAS*, *EPO*, *CXCL12*, *TNFRSF11B*, *KITLG*, *CCL4*, *CCL25. IL7*, *CXCL13, IL15*, *INFG*, *VEGF*A) and females (*TIMP2*, *PLAT*, *FAS*, *IL15*, *INFG*) during aging in aVAT (Figure 3). Based on the pattern of the clusters throughout time, the deconvolution analysis, and the SASP expression, these data indicate marked changes in aVAT’s cellularity during aging, which was concurrent with “inflammaging” in both males and females. 

### 3.2. Prediction of an Age-Associated Omentum-Liver Connectome 

Considering that aging results in widespread changes in genes encoding secreted proteins in aVAT, and the fact that the aVAT secretome is drained by the portal system, we hypothesized that the age-induced alterations in the aVAT secretome would have a predicted impact on the liver. Therefore, we sought to determine the genes that encode proteins that are secreted and potentially interact with a reported receptor expressed in the liver. In that sense, based on the genes found in clusters A, B, C and I, the use of protein–protein interaction databases, manual curation, and expression levels, we generated what we called the age-associated omentum-liver connectome, resulting in 11 genes (*ADIPOQ*, *LPL*, *FCN2*, *IL1RN*, *TF*, *IGF1*, *VEGFA*, *CXCL10*, *ASIP*, *LIPC*, *INFG)* and 13 predicted receptors (*ADIPOR1*, *ADIPOR2*, *MGRN1*, *SDC1*, *TFR2*, *F11R*, *IL1R*, *SDC4*, *LRP1*, *IFNGR1*, *INSR*, *ITGB1*, *GPC1*).

**Figure 4:**
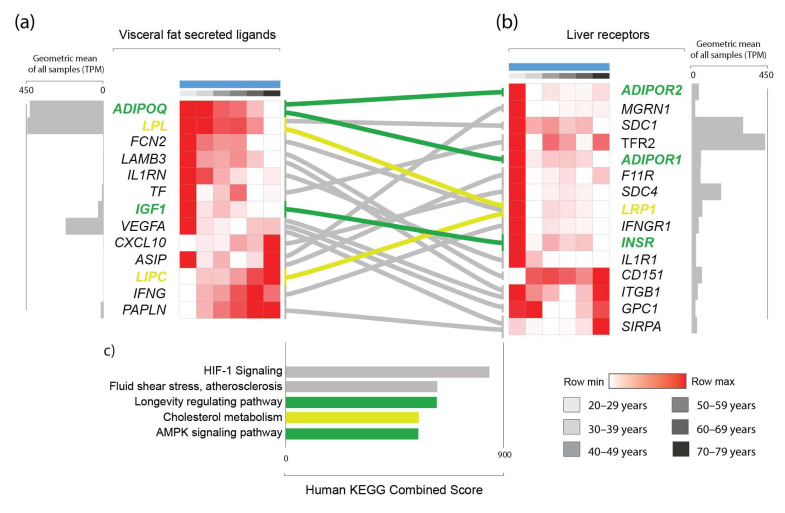
Predicted connectome between visceral fat (**a**) and the liver (**b**) in males, including predicted visceral fat secreted ligands and their experimentally demonstrated liver receptors. Bar graph demonstrating enrichment scores from the Kyoto Encyclopedia of Genes and Genomes (**c**), as provided by Enrichr [28], revealed aging- and metabolic disease-related pathways (**c**). Only genes that are part of the “Longevity regulating”, “Cholesterol metabolism”, and “AMPK signaling” pathways are highlighted in green and yellow, respectively. Other genes appear in multiple other pathways and are displayed in grey without highlighting. Row values are normalized by Z-score, so minimum and maximum values are row relative. The geometric mean of all samples of transcripts per million (TPM) is displayed to reflect the absolute nonrelative expression of the gene in the tissue compared with other genes.

In this connectome, we found that the “HIF-1 Signaling”, “Cholesterol metabolism”, “Fluid Shear and Atherosclerosis”, “Proteoglycan in cancer”, “Longevity regulating pathway”, “AMPK signaling pathway,” and “Cytokine-cytokine receptor interaction” pathways were significantly enriched (FDR < 0.05) (Figure 4).

We have also found that mutations in many of the genes in this connectome have been previously associated with longevity, aging, and cardiometabolic factors in genome-wide association studies (GWAS) (Material S6). For example, increased adiponectin (*ADIPOQ*) mutations that potentiate its activity are associated with longevity; interleukin 1 receptor antagonist (*IL1RN*), transferrin (*TF*), transferrin receptor 2 (*TFR2*) vascular endothelial growth factor A (*VEGFA*), agouti signaling protein (*ASIP*), mahogunin ring finger 1 (*MGRN1*) and syndecan 4 (*SDC4*) mutations are all associated with cholesterol levels. Moreover, *ASIP*, *IGF1*, *VEGFA*, *SDC1*, interferon-gamma (*IFNG)*, adiponectin receptor 1 (*ADIPOR1)*, and interferon-gamma-receptor-1 (*IFNGR1)* mutations are associated with various body composition measurements such as body mass index, abdominal circumference, and waist-to-hip ratio. Finally, mutations of many of these genes are also associated with healthy aging or an increased lifespan, such as *ADIPOQ*, *VEGF*, *IFNG*, *SDC4*, and *LDL* receptor-related protein 1 (*LRP1).*

## 4. Discussion

Here, we used a computational approach to study the effects of aging on visceral fat in humans and its potential underlying consequences on the liver through an age-induced visceral fat-liver connectome. Particularly, we have demonstrated that specific clusters of genes are progressively changing throughout aging in aVAT. In our analyses, DEGs appear more frequently at an early age in the visceral fat of men than in women. Moreover, considering all age groups, the total number of DEGs comparing males and females was also different (708 vs. 108, respectively). We observed that the cellular remodeling was remarkable in males. Moreover, changes in SASP-related genes in aVAT due to aging were observed, although these alterations are less noticeable in women. We cannot rule out that technical reasons may explain, to some extent, these observations. First, the total amount of data analyzed among males and females was different (216 for the male group vs. 116 for the female group), thus reflecting differences within the age groups in terms of sample size and statistical power. Lastly, we were not able to control the effect of contraceptive usage or other hormonal treatment, body mass index, or any clinical condition in our analyses due to the lack of available information. Of note, it is important to mention that women mainly store fat in gluteal-femoral subcutaneous adipose tissue, but inherent changes from pre- to postmenopausal stages (~42 to ~52 years) are marked by the accumulation of fat in the visceral compartment [40]. Interestingly, this period matches our data regarding the age when we start to observe DEGs in aVAT from women. Taken together, these results indicate sexual dimorphism in the response of visceral fat to aging. These differences may be related to the protective effect of female gonadal hormones on adipose tissue in the context of aging. In that sense, the proposed omentum-liver connectome was built based on our male cohort due to its increased robustness.

Aging is marked by a decline in adipose tissue functioning and regeneration, which in turn contribute to metabolic impairment at the organismal level. At the adipose tissue level, lower adipogenic potential, accumulation of hypertrophic adipocytes, fibrosis, accumulation of senescent cells, and inflammation are features that have been described by studies conducted in mice and humans as a consequence of aging [13,16,17,18,20,21]. Accordingly, our analysis predicted a lower preadipocyte abundance in men, although this is less clear in women. Furthermore, a decrease in adipocyte abundance was predicted in both sexes. Moreover, our data also demonstrated higher fibroblasts scores during the aging process in males, although this is less noticeable in women. Therefore, our findings are aligned with the literature. Adipose tissue cellular remodeling induced by aging is thought to not be merely restricted to the mesenchymal lineage, as it also includes alterations in the myeloid and lymphoid lineages, according to recent studies carried out in mice and humans [14,17,21]. Our deconvolution analysis predicted a decrease in the scores of monocytes, natural killer cells (NKT), and neutrophils in men, while only a decrease in NKT cells was predicted in women. On the other hand, we observed an increase in CD4^+^ and CD8^+^ T cells in men and CD8^+^ T in women. We did not observe alterations in B cells in both sexes. We acknowledge that our work relies on in silico predictions from transcriptomic data, and we were unable to validate our findings. However, these observations are supported by other studies. Single-cell RNA sequencing analysis performed on young and old mice shows significant alterations in immune cells within adipose tissue due to aging. For instance, lower NKT cells and myeloid cells decreased, while B cells and T cells increased [17]. The increase in T cells within adipose tissue due to aging is also confirmed by experimental evidence in mice [15]. Interestingly, Trim and colleagues demonstrated, in humans, that CD4^+^ and CD8^+^ T cells per gram of subcutaneous adipose tissue are higher in old donors [21].

Inflammation is considered a hallmark of aging. Indeed, our data show it to be the case in human visceral adipose tissue. Cellular senescence is a process that is present in human adipose tissue-derived stem cells during aging [20]. Moreover, evidence demonstrates that adipocyte senescence contributes to adipose tissue inflammation through SAPS [41]. We observed that terms involved in inflammatory processes were retrieved because of our enrichment analysis in the age-associated modules. Moreover, we observed that expression of SASP-related genes was increased within adipose tissue due to aging. Hence, our data support the notion that cells within the tissue acquire a pro-inflammatory secretome. As we evaluated transcriptomic data from whole fragments of aVAT, our analyses were not able to define which cellular fraction—adipocytes or stromal vascular fraction—is responsible for the alterations in the enriched terms of the inflammatory pathways and SASP-related genes. However, irrespective of the differential response of aging on specific cell types, if the aVAT is drained by the portal vein, then the total changes in the secretion output of aVAT components would reflect in the portal system. This provided us with a rationale to investigate the connectivity between the age-dependent aVAT secretome and its respective receptors in the liver, thereby generating what we named the age-induced omentum-liver connectome. Our analyses predicted a potential connectivity through genes known to mediate the endocrine function of the adipose tissue and have established activity in the liver (e.g., *ADIPOQ*, *IGF1*, *INFG*, *CXCL10*, *VEGFA*, *IL1RN*, *LPL*). In humans, adiponectin/ADIPOQ signaling protects against metabolic diseases [42]. Moreover, adiponectin serum levels are higher in exceptionally long-lived humans when compared to non-long-lived individuals [43]. *Adipoq* null mice have a slightly decreased lifespan and manifest premature metabolic dysfunction, while mice overexpressing *Adipoq* have a prolonged health span [44]. Consistently, we observed that *ADIPOQ* is reduced with aging in human visceral adipose tissue. As seen in our network analysis (Figure S4), adiponectin was predicted to bridge the interaction between genes that decrease expression (clusters A, B, and C) and those that increase expression (cluster I) through their interactions with *IFNG*. Indeed, IFNG was shown to inhibit *ADIPOQ* expression and differentiation in adipocytes [45]. Simultaneously, ADIPOQ can reduce *IFNG* and T cells in obese mice [46], indicating a “dual carriageway”. Based on our methods and results, it is not possible to determine which of those two comes first in the aging process. The hypothesis of a vicious cycle cannot be discarded. In addition to this, our findings on *IGF1*, *INFG*, *CXCL10*, *VEGFA*, *IL1RN*, and *LPL* and their predicted interactions with their receptors in the liver are all of interest because unfavorable metabolic health is expected with the aging of the organisms. Thus, it would be reasonable to speculate that this altered connectivity between visceral fat and the liver may be underlying a worse metabolic phenotype in the elderly. Furthermore, our analyses detected known adipose tissue modulators of metabolic function, which strengthens the predictions regarding unexpected genes with a role in the context of age-related diseases through the visceral fat–liver axis (e.g., *FCN2* and *TF*).

Our analyses predicted the interaction between aVAT-produced FCN2 and its receptor LRP1 in the liver upon aging. Previous GWAS studies revealed that *LRP1* mutations affect aging, fasting glucose levels, healthy aging, and cholesterol levels (Material S6). We observed that *FCN2* expression progressively decreases due to age in aVAT, which may influence the liver through LRP1 (low-density lipoprotein receptor-related protein 1). The *FCN2* gene encodes the protein ficolin-2 (FCN2), which binds to specific pathogen-associated molecular patterns on the pathogen surface, thereby playing a role in innate immunity through the complement lectin system as an opsonin. FCN2 is detected in the serum of healthy individuals with great variability (~320–8000 ng/mL) [47]. According to the human protein atlas, FCN2 is also expressed in the stromal vascular fraction of adipose tissue [26,27]. FCN2 has a high affinity for N-acetylated and neutral carbohydrates, and its interaction with LRP1 has been experimentally demonstrated [48]. Regarding its (patho)physiological role, FCN2 serum levels are induced by the dengue virus infection in humans, are positively correlated with platelet levels, and are negatively correlated with aspartate transaminase (AST) [49]. Furthermore, FCN2 may suppress hepatocellular carcinoma migration and invasion, as demonstrated by a study conducted on mice [50]. Considering that response to infection decreases during aging and hepatocellular carcinoma is also an age-related disease, our findings with regard to *FCN2* is of potential relevance. Moreover, the function of *FCN2* in adipose tissue is not known. 

Our omentum-liver connectome also predicted a decrease in *TF* expression in aVAT and disrupted connectivity between this gene and its receptor *TFR2* in the liver upon aging. More importantly, overlaps between GWAS studies revealed that mutations in both TF and its receptor are associated with cholesterol levels (Material S6). *TF* encodes the glycoprotein transferrin, which is involved in iron transport and distribution. Serum TF is mainly produced and secreted to the circulation by the liver. TF binds to its receptors TFR1 and TRF2, thus transporting iron to tissues. The receptor TFR2 is expressed mainly in hepatocytes [51]. According to experimental evidence, *TF* is also expressed in adipose tissue, more specifically in mature adipocytes and not in the stromal vascular fraction [52] Hence, the experimental evidence available regarding the expression of *TF* and its receptor TFR2 supports our prediction. Notably, iron metabolism is linked to metabolic disturbances [53]. However, whether iron metabolism in adipose tissue is related to metabolic disturbances is less understood. The term “Ferroptosis,” which is a kind of programmed cell death associated with iron, the accumulation of lipid peroxides, and *TF,* was enriched in cluster A (Figure 1). Evidence has demonstrated that lower *TF* expression in adipocytes decreases intracellular iron levels and disrupts insulin sensitivity. Importantly, lower *TF* expression in the subcutaneous adipose tissue of humans is associated with reduced systemic insulin sensitivity. Moreover, this negative correlation between *TF* expression and insulin sensitivity does not occur in the liver and muscle [52]. Therefore, our findings that *TF* expression decreases with age in aVAT are relevant in terms of the potential pathophysiological relationship with age-related diseases, mainly metabolic ones.

In conclusion, we demonstrated that aging promotes widespread alterations in gene expression within aVAT. These alterations in the transcriptome underlie cellular remodeling and changes in SASP-related genes, thereby potentially affecting the adipose tissue’s secretome and disrupting the connectivity between aVAT and liver. Our data provide a broad landscape of human visceral fat aging. Furthermore, this work provides a resource for the community, as it can be used to guide efforts toward a deeper investigation of adipose tissue genes that control liver function in the context of aging. 

## Data Availability

The data that support the findings of this study are available in GTEx Portal at https://gtexportal.org/home/, accessed on 20 January 2020, DOI: 10.1038/ng.2653. These data were derived from the following resources available in the public domain: https://gtexportal.org/home/datasets.

## Supplementary Materials

The following supporting information can be downloaded at: https://www.mdpi.com/article/10.3390/biomedicines11051446/s1. Supplementary files of the analyses are described as follows: Supplementary Material S1: Male DEGs; Supplementary Material S2: Female DEGs; Supplementary Material S3: Gene clusters; Supplementary Material S4: Enrichment results of each cluster; Supplementary Material S5: Deconvolution results of each sample; Supplementary Material S6: GWAS results of the connectome; Supplementary Material S7: Eigenvector centrality of the gene networks; Supplementary Table S1: Comparison of protein-protein interaction enrichment p-values of our clusters with randomly generated ones; Figure S1: Population and DEG distribution; Figure S2: Heatmap of mean expression of DEGs and enrichment of their clusters; Figure S3: Normalized mean expression of each cluster, distributed by age and sex; Figure S4: Network analysis of clusters A, B, C and I.
