# Supplementary material for: The Transcriptomic Landscape of Age-Induced Changes in Human Visceral Fat and the Predicted Omentum-Liver Connectome in Males
Source: Biomedicines. 2023 May 14;11(5):1446. doi: 10.3390/biomedicines11051446 (PMC10216224; doi:10.3390/biomedicines11051446)
Supplement: Supplementary file 1 [file biomedicines-11-01446-s001.zip › biomedicines-2237156-figure S1-S4.pdf]

biomedicines

MDPI

Supplementary Figures

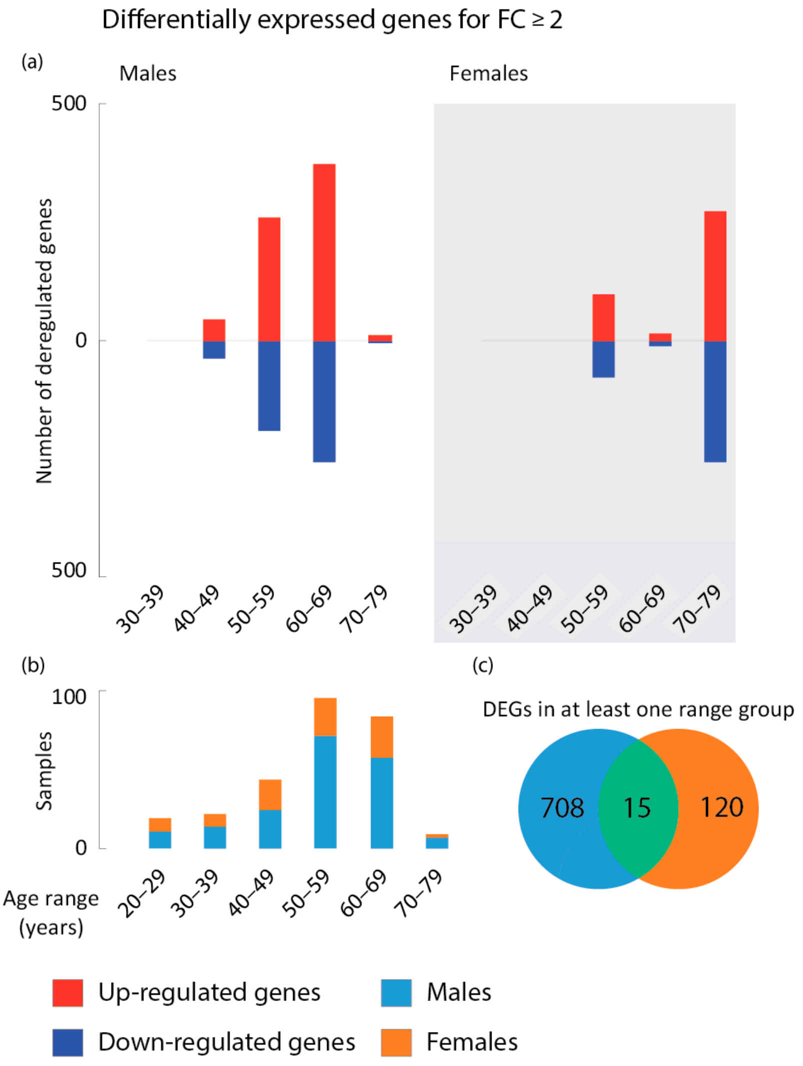

Figure S1. Differentially expressed genes (DEGs) of each age range from the oldest group to the youngest group (**a**). The youngest group (20–29 years old) was used as a common reference using the Genotype-Tissue Expression database. Differences were only observed for the proposed cutoff (FC ≥ |2|, FDR < 0.05) after the 40–49 age range for males and 50–59 age range for females. Comparatively, there were fewer DEGs in 70–79-year-old males, and the opposite occurred in 70–79-year-old females. This could be due to smaller sample sizes of 70–79-year-olds (**b**). Quantitative representation of common and specific DEGs for males, females, and between the groups are represented by Venn diagram (**c**).

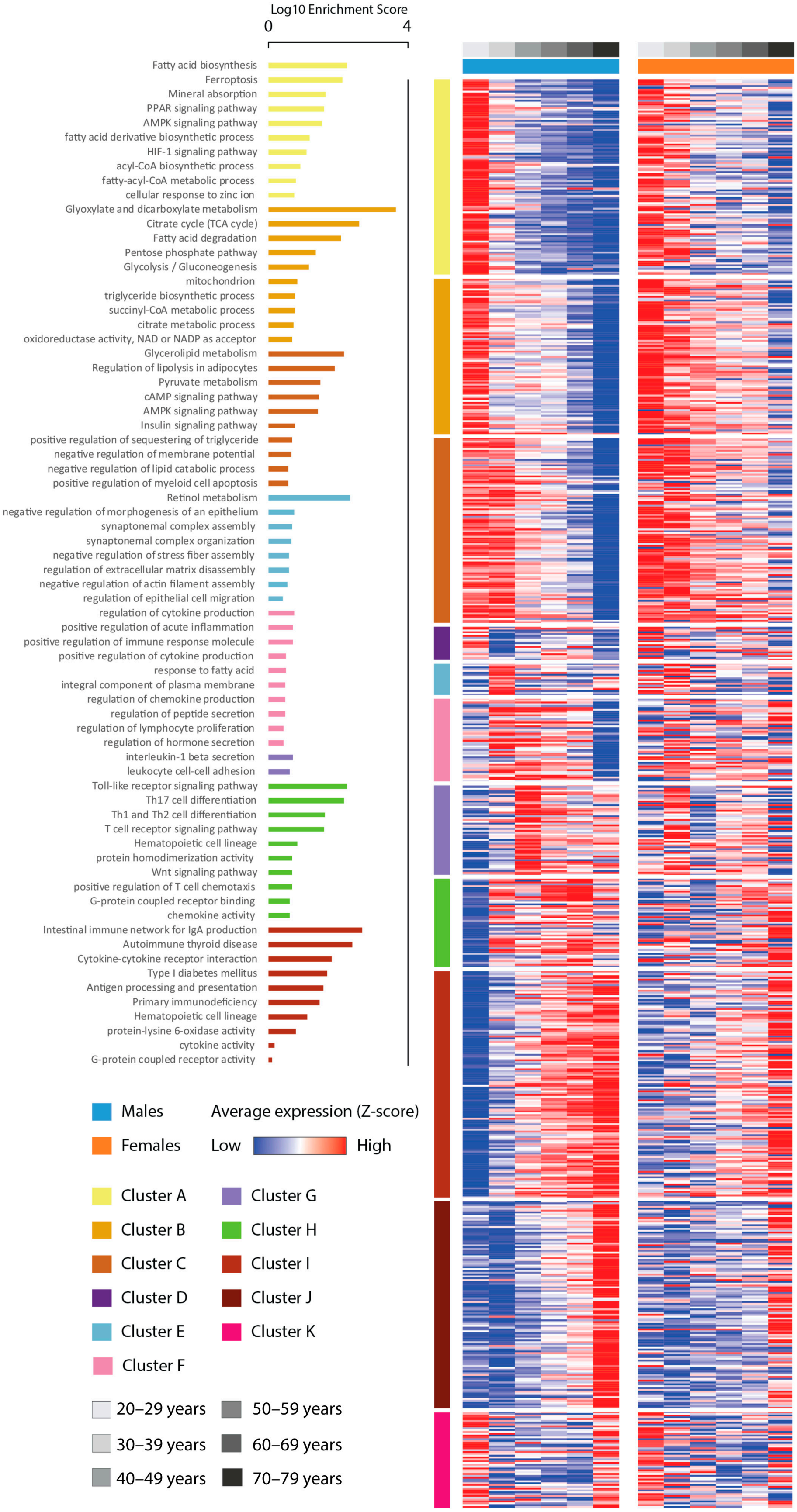
Log10 Enrichment Score
0
4
Fatty acid biosynthesis
Ferroptosis
Mineral absorption
PPAR signaling pathway
AMPK signaling pathway
fatty acid derivative biosynthetic process
HIF-1 signaling pathway
acyl-CoA biosynthetic process
fatty-acyl-CoA metabolic process
cellular response to zinc ion
Glyoxylate and dicarboxylate metabolism
Citrate cycle (TCA cycle)
Fatty acid degradation
Pentose phosphate pathway
Glycolysis / Gluconeogenesis
mitochondrion
triglyceride biosynthetic process
succinyl-CoA metabolic process
citrate metabolic process
oxidoreductase activity, NAD or NADP as acceptor
Glycerolipid metabolism
Regulation of lipolysis in adipocytes
Pyruvate metabolism
cAMP signaling pathway
AMPK signaling pathway
Insulin signaling pathway
positive regulation of sequestering of triglyceride
negative regulation of membrane potential
negative regulation of lipid catabolic process
positive regulation of myeloid cell apoptosis
Retinol metabolism
negative regulation of morphogenesis of an epithelium
synaptonemal complex assembly
synaptonemal complex organization
negative regulation of stress fiber assembly
regulation of extracellular matrix disassembly
negative regulation of actin filament assembly
regulation of epithelial cell migration
regulation of cytokine production
positive regulation of acute inflammation
positive regulation of immune response molecule
positive regulation of cytokine production
response to fatty acid
integral component of plasma membrane
regulation of chemokine production
regulation of peptide secretion
regulation of lymphocyte proliferation
regulation of hormone secretion
interleukin-1 beta secretion
leukocyte cell-cell adhesion
Toll-like receptor signaling pathway
Th17 cell differentiation
Th1 and Th2 cell differentiation
T cell receptor signaling pathway
Hematopoietic cell lineage
protein homodimerization activity
Wnt signaling pathway
positive regulation of T cell chemotaxis
G-protein coupled receptor binding
chemokine activity
Intestinal immune network for IgA production
Autoimmune thyroid disease
Cytokine-cytokine receptor interaction
Type I diabetes mellitus
Antigen processing and presentation
Primary immunodeficiency
Hematopoietic cell lineage
protein-lysine 6-oxidase activity
cytokine activity
G-protein coupled receptor activity
Males
Females
Average expression (Z-score)
Low
High
Cluster A
Cluster B
Cluster C
Cluster D
Cluster E
Cluster F
Cluster G
Cluster H
Cluster I
Cluster J
Cluster K
20–29 years
30–39 years
40–49 years
50–59 years
60–69 years
70–79 years

Figure S2. Heatmap of the mean expression of differentially expressed genes (DEGs) in at least one age group clustered with K means = 11The expression of some genes progressively increases with aging at different rates (clusters A, B, and C), while others decrease (clusters I and J). In addition, some do not form gradients and have overall lower enrichment scores (D, E, F, G, H, J, and K).

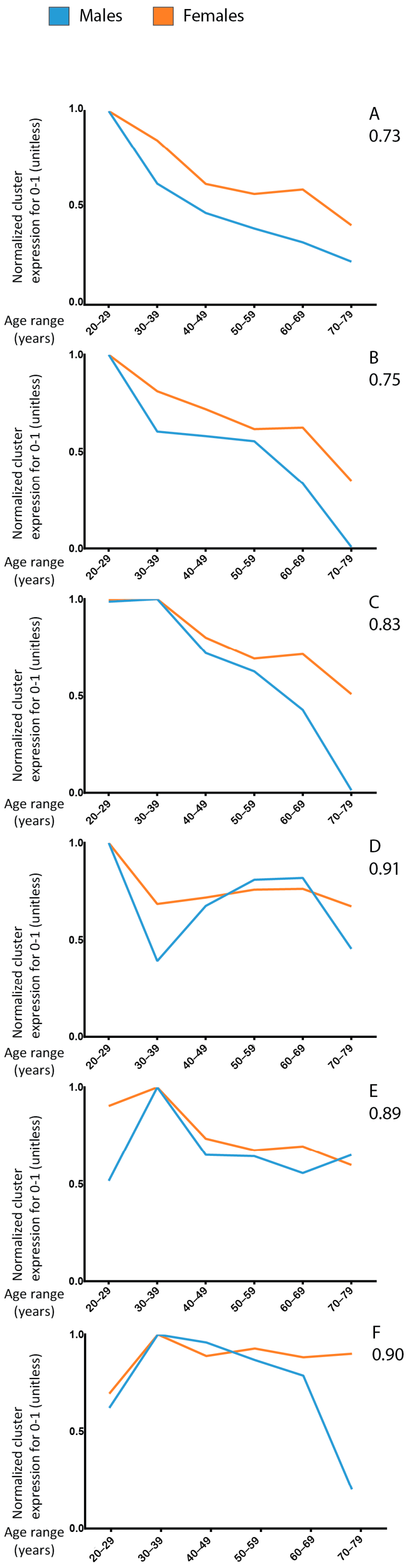

Figure S3. Unitless (0, 1) and normalized mean expression of each cluster for males and females and the ratio of the AUC between males and females (upper right corner of each graph). The closer to 1, the higher the similarity between males and females. In clusters A, B and C we can see a downward trend, while in cluster I and J, a growing trend.

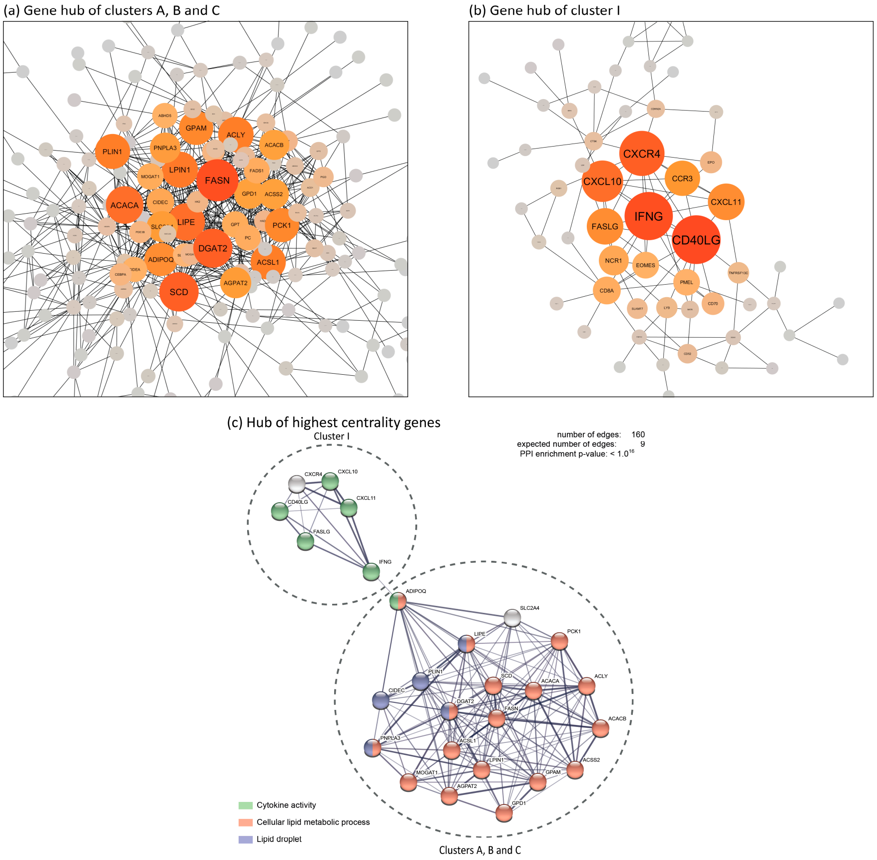

Figure S4. Gene–gene interaction analysis concerning physical protein–protein expression, text mining, and co-expression connectivity between clusters. Node sizes correspond to eigenvector centrality (**a**) in genes whose expression increase gradually with age and (**b**) decrease with age. Finally, (**c**) the interaction between the genes with the highest centrality of all clusters.
