# Supplementary material for: The Transcriptomic Landscape of Age-Induced Changes in Human Visceral Fat and the Predicted Omentum-Liver Connectome in Males
Source: Biomedicines. 2023 May 14;11(5):1446. doi: 10.3390/biomedicines11051446 (PMC10216224; doi:10.3390/biomedicines11051446)
Supplement: Supplementary file 1 [file biomedicines-11-01446-s001.zip › Table S1.pdf]

Supplementary Table 1

Cluster s	PPI Enrichment score	Number of genes	Random gene set	PPI Enrichment score	Number of genes
A	**1.08E-09**	108	1	0.499	50
B	**6.44E-05**	86	2	0.898	50
C	**< 1.0e-16**	102	3	0.197	100
D	0.448	19	4	0.675	250
E	0.148	18	5	0.5	250
F	**0.00741**	46	6	0.871	350
G	**0.000805**	50	7	0.447	350
H	**1.29E-07**	49	8	0.733	400
I	**1.60E-12**	125	9	0.265	800
J	**0.00271**	114	10	**0.03**	800
K	**0.0159**	54	11	0.808	800

Cluster quality test performed on STRINGDB. Clusters had very significant protein-protein interaction p-values (PPI) compared to randomly selected genes.
